# New Insights into the Biology and Diagnosis of Splenic Marginal Zone Lymphomas

**DOI:** 10.3390/curroncol28050297

**Published:** 2021-09-06

**Authors:** Marie Donzel, Lucile Baseggio, Juliette Fontaine, Florian Pesce, Hervé Ghesquières, Emmanuel Bachy, Aurélie Verney, Alexandra Traverse-Glehen

**Affiliations:** 1 Institut de pathologie multi-sites, Hôpital Lyon Sud, Hospices Civils de Lyon, 69310 Pierre Bénite, France; marie.donzel@chu-lyon.fr (M.D.); juliette.fontaine@chu-lyon.fr (J.F.); florian.pesce@chu-lyon.fr (F.P.); 2 Laboratoire d’hématologie, Hôpital Lyon Sud, Hospices Civils de Lyon, 69310 Pierre Bénite, France; lucile.baseggio@chu-lyon.fr; 3 INSERM-Unité Mixte de Recherche 1052 CNRS 5286, Team “Clinical and Experimental Models of Lymphomagenesis”, UCBL, Cancer Research Center of Lyon, Université Lyon, 69001 Lyon, France; herve.ghesquieres@chu-lyon.fr (H.G.); emmanuel.bachy@chu-lyon.fr (E.B.); aurelie.verney@univ-lyon1.fr (A.V.); 4 Service d’hématologie, Hôpital Lyon Sud, Hospices Civils de Lyon, 69310 Pierre Bénite, France

**Keywords:** splenic marginal zone lymphoma, review, genetic, molecular

## Abstract

Splenic marginal zone lymphoma (SMZL) is a small B-cell lymphoma, which has been recognized as a distinct pathological entity since the WHO 2008 classification. It classically presents an indolent evolution, but a third of patients progress rapidly and require aggressive treatments, such as immuno-chemotherapy or splenectomy, with all associated side effects. In recent years, advances in the comprehension of SMZL physiopathology have multiplied, thanks to the arrival of new devices in the panel of available molecular biology techniques, allowing the discovery of new molecular findings. In the era of targeted therapies, an update of current knowledge is needed to guide future researches, such as those on epigenetic modifications or the microenvironment of these lymphomas.

## 1. Introduction

Splenic marginal zone lymphoma (SMZL) is a small B-cell lymphoma, which was first described in 1992 and has been recognized as a distinct pathological entity since the WHO 2008 classification. It accounts for 2% of lymphoid malignancies [1] and is mostly clinically characterized by the progressive appearance of a pain-causing splenic enlargement associated with the perturbation of complete blood count, cytopenia, or appearance of autoimmune disorders such as autoimmune haemolytic anemia (AIHA). It usually occurs at a mean age of 67 years and classically presents an indolent evolution, with survival rates at 10 years ranging from 67% to 95% [2]. Even so, a third of patients progress rapidly and require quite aggressive treatments, such as immuno-chemotherapy (rituximab [2,3] sometimes associated with CHOP) or splenectomy, with all the side effects involved (notably infectious complications after splenectomy). In addition, Perrone et al. showed that after 10 years of follow-up, only 17% of patients would not require any treatment [2]. The clinical and biological intergruppo Italiano Limfomi (ILL) score and the hemoglobin-platelet-LDH-lymphadenopathy (HPLL) scores have been proposed to identify patients at risk of relapse but remained controversial, depending on the studied population [4,5] and used aspecific blood markers. The only currently admitted adverse clinical prognostic factors include a large tumor mass and a poor general health status. These data demonstrate a significant impact on public health costs and highlight the lack of knowledge on the pathophysiology of this lymphoma and its prognostic markers. In recent years, discoveries on pathophysiology, thanks to advances in molecular biology, have multiplied, but systemic reviews on this subject are rare [6]. This article reviewed the recent advances in SMZL physiopathology based on new updates on biological findings, histological features, and molecular biology. 

## 2. Results

### 2.1. Clinical Presentation and Current Care

SMZL appears more often in women (with a sex ratio of 0.44) [7], between 59 and 65 years old. Clinical warning signs are splenomegaly (often revealed by abdominal pain or heaviness or even discovered after a traumatic spleen rupture) and lymphocytosis, sometimes associated with cytopenias. Lymphadenopathies are rare in non-transformed cases and classically localized in the hile of the spleen. B symptoms are uncommon but can be observed. Bone marrow involvement is almost constant (83–100%), while peripheral blood dissemination is variable (29–75%) [6]. Autoimmune disorders (autoimmune hemolytic anemia, cryoglobulinemia, idiopathic thrombocytopenic purpura, and others) are frequent, as well as monoclonal components (mostly Immunoglobulin M (IgM), classically with levels of <3 g/dL in about 50% of the cases) [8].

Splenectomy is nowadays much less performed in the treatment of SMZL, because there are alternatives, for both diagnosis and treatment. Splenectomy remains the reference concerning the diagnosis. However, it can also be established, thanks to the cytological analysis and flow cytometry of peripheral blood or bone marrow aspirate [9]. Regarding treatment, management differs, depending on whether patients are symptomatic or not. For asymptomatic patients, active surveillance is now recommended with regular follow-up consultations [9]. In cases of isolated autoimmune disorders, it is recommended to treat them specifically, for example, using rituximab therapy alone (375 mg/m^2^; 4-to-8-week doses) [9]. Concerning symptomatic patients and according to the 2020 ESMO practical guidelines, criteria for initiating treatment are the presence of progressive or symptomatic splenomegaly and/or cytopenia (hemoglobin: <100 g/L; platelets: <80 g/L; neutrophils: <1 g/L). In these cases, therapeutic options are splenectomy and rituximab, or conventional chemotherapy such as CHOP (rituximab, cyclophosphamide, doxorubicin, and vincristine). Response to splenectomy with correction of cytopenia occurs in approximately 90% of patients [10] and has been associated with statistically significantly better overall survival and progression-free survival than non-splenectomized patients in a recent study [11].

Nevertheless, SMZL patients are often elderly and have surgical risks. Since the approval of rituximab, the treatment of such patients with the anti-CD20 antibody alone or in combination with chemotherapy has shown remarkable responses [10]. Concerning refractory or relapsing cases, ibrutinib has shown exciting results in relapsed/refractory SMZL treated with prior rituximab, with an overall response rate of 62% [12].

### 2.2. Diagnosis

#### 2.2.1. Cytology, Immunophenotype, and Pathology

**Figure 1:**
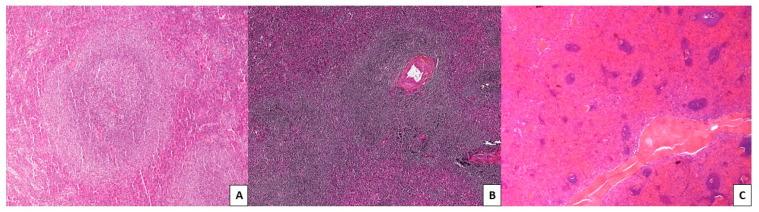
Histological patterns of splenic marginal zone lymphoma (SMZL; magnification: ×10). (**A**) Biphasic pattern: small lymphocytes surround and replace the white pulp follicles and merge with a peripheral zone of larger marginal zone (MZ)-like cells, including scattered transformed blasts, giving the characteristic biphasic pattern. (**B**) Monophasic pattern: cases lack a central core of smaller lymphocytes and have a monophasic pattern. (**C**) Atrophic pattern, with an atrophic white pulp and few infiltrating cells in the red pulp.

Cytology shows a mixture of heterogeneous cells, including small lymphocytes (with a round nucleus and compact chromatin, often condensed in small irregular clumps) admixed with lymphoplasmacytic cells. They are sometimes associated with villous lymphocytes in a tiny percentage and with shorter projections than other splenic lymphomas, particularly splenic diffuse red pulp lymphoma (SDRPL) or hairy cell leukemia (HCL). In the bone marrow, SMZL infiltration is classically nodular and intrasinusoidal but less frequently interstitial [6]. However, intrasinusoidal infiltration is not specific for SMZL and can be observed in other small B-cell lymphomas such as mantle cell lymphoma (MCL) or chronic lymphocytic leukemia (CLL). In the spleen, SMZL is characterized by an expansion of the splenic white pulp with the infiltration of the red pulp. As illustrated in Figure 1, three patterns can be observed: two nodular patterns and an atrophic pattern; (i) the more frequent is the biphasic pattern, in which small lymphocytes surround and replace the germinal centers of the white pulp, giving it a cockade-like appearance; (ii) the second nodular pattern, so-called monophasic pattern, also present an enlargement of the white pulp with a more homogeneous appearance; (iii) the third pattern displays an atrophic white pulp with few infiltrating cells in the red pulp. The red pulp infiltration can either be nodular, diffuse, mixed or even solely diffuse. Concerning differential diagnosis with other entities of marginal zone lymphomas (MZL) such as mucosa-associated lymphoid tissue (MALT lymphoma) or nodal MZL (NMZL), in cases where the disease is still at an early stage, it is essentially the clinical presentation that will be useful (location, splenic, and/or lymph node involvement). In patients with generalized disease at diagnosis, differential diagnosis with a small B-cell lymphoma involving the spleen can be challenging. In these cases, molecular biology can help clarify the diagnosis, mainly by finding the characteristic abnormalities of SMZL, as described further below [13,14,15,16,17,18].

In some cases, lymphoma cells may show a variable degree of plasmacytic differentiation, up to 30% of tumoral cells, associated with a monotypic light chain expression, thus making the differential diagnosis with lymphoplasmacytic lymphoma (LPL) sometimes tricky. However, clinical presentation and molecular biology are different (see below). In 10% to 20% of cases, a histological transformation into a high-grade B-cell lymphoma, such as diffuse large B-cell lymphoma, can occur [6].

**Figure 2:**
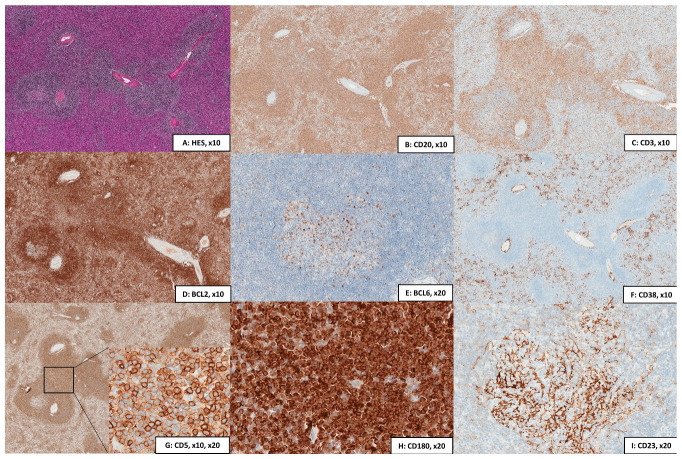
Immunohistochemical patterns of SMZL. (**A**) Hematoxylin-eosin-safran (HES) staining showing a nodular (monophasic pattern) with the enlargement of the white pulp (magnification: 10×); (**B**) CD20 staining showing the hyperplasia of the white pulp with a variable involvement of the red pulp (magnification: 10×); (**C**) CD3 staining by immunohistochemistry also showing variable numbers of CD3 + T cells (magnification: 10×); (**D**) BCL2 showing that SMZL cells overexpressed BCL2 (magnification: 10×); (**E**) BCL6 expressed in residual germinal centers but not on tumoral cells (same staining using CD10; magnification: 20×); (**F**) CD38 staining highlighting a large number of associated plasma cells (magnification: 10×); (**G**) CD5 staining of a CD5 + SMZL, with weaker staining on B-cells than on T cells of the microenvironment (magnifications: 10× and 20×); (**H**) CD180 staining in SMZL (magnification: 20×); strong membranous and cytoplasmic positivity with perinuclear accentuation; (**I**) CD23 staining highlighting a nodular meshwork of follicular dendritic cells in the white pulp (magnification: 20×).

The immunohistochemistry (Figure 2) shows a mature B phenotype with the expression of CD20, and CD79, without cyclin D1, CD10, or BCL6. The immunohistochemistry with CD23 classically highlights a nodular network of follicular dendritic cells in the white pulp. By flow cytometry, cells are CD24+, CD27+, and FMC7+. They are classically stained by CD22 and CD11c but less bright than other splenic lymphomas (SDRPL or HCL). The CD123 is negative, and the CD103 may be dimly positive in rare cases. With both techniques, cases are usually negative for Annexin A1 and CD25. The addition of CD180 in the flow cytometry and immunohistochemistry panel has made it possible to better classify these lymphomas with sensitivity and specificity evaluated at least at 75% and 90%, respectively [19,20], making CD180 a helpful immunologic marker in MZL. Moreover, the intensity of CD180 staining may favor a splenic origin of the lymphoma, as SMZL and SDRPL display particularly high levels of CD180 expression in flow cytometry [21]. In rare cases (10–15%), tumoral cells can be positive for CD23 or CD43 [22,23]. A group of CD5+ SMZL has been described, distinguished by a higher lymphocytosis and diffuse bone marrow infiltration [24], but the simultaneous expression of CD5 and/or CD43 with CD23 is rare. In these cases, chronic lymphocytic leukemia (CLL) should be excluded. CD200 expression constitutes a major immunological marker in the differential diagnosis of CD5+ small B-cell lymphomas; for example, mantle cell lymphoma is CD200-negative (in contrast to SMZL) and also in small B-cell lymphomas with villous cytoplasm; SDRPL is dimly CD200-positive, whereas HCL is strongly CD200-positive [25]. Proliferative index using Ki67/Mib1 is generally low, with a characteristical pattern (high in the germinal center and the marginal zone and low elsewhere) [6].

#### 2.2.2. Cytogenetics

Unlike other B-cell lymphomas, few recurrent abnormalities are observed. The most frequent are 7q deletions (30–40%), likely in the loci 7q32 to 7q35 [26,27,28], or trisomies of chromosomes 3 or 12 [13,29], and even 1q, 8q, 18, or 6q deletions [30]. The 7q deletion is seen much more frequently in SMZL than similar B-cell neoplasms, and thus, it has even been proposed as a primary diagnostic marker. Some authors suggest that it may be a causative event rather than a simple pro-survival signal and that the 7q31–32 deletion may be associated with a typical IgM+, IgDdim, CD5−, CD10−, or CD23− immunophenotype and inversely correlated with trisomy 18 [27,31]. The gene(s) targeted by the 7q deletion and the precise location of the specific deletion area remain unknown, even if numerous studies have tried to highlight it, using high-resolution array comparative genomic hybridization or studying miRNA (see below) [26,27,28,29,32,33]. Regarding its prognostic impact, Algara et al. also associated the 7q31 deletion with poor overall survival [34], but this impact remains controversial [27].

### 2.3. Molecular Biology

#### 2.3.1. Mutational Status of Immunoglobulin Heavy Chain (IGVH) Genes

It is expected that SMZL derives from a marginal zone B-cell with possible previous antigen exposure. The rearrangements of the immunoglobulin genes found in 30% of SMZL cases may suggest that this tumor derives from a highly selected B-cell population [34,35,36,37]. The initial hypothesis was, indeed, that SMZL derived from marginal zone B cells due to a histological marginal zone differentiation in splenic specimens [34,38] and the use of a limited repertoire, with a preferential usage of specific IGHV genes, such as IGHV1-2*04 (31%), IGHV3-23 (8%), and IGHV4-34 (13%). In addition, approximately 10% of cases express B-cell receptors (BCRs) with quasi-identical IGHV sequences, including the antigen-binding site, strongly suggesting that antigen selection might contribute to the lymphomagenesis of SMZL [36,39]. Moreover, Warsame et al. reported that SMZL with an HV1-2 rearrangement is not associated with hepatitis C virus infection and produced poly- and self-reactive antibodies [40]. Their results indicate that some SMZL arise from poly-reactive B cells, a subset of marginal zone B cells important in the immunologic defense against infection, raising the possibility that a “super-antigen” is involved in its lymphomagenesis. Numerous studies confirm the presence of a biased repertoire with a preferential usage of certain IGHV genes and suggest that some specific antigens could trigger the lymphomagenesis of SMZL [31,34,37,39]. Some SMZL may then develop themselves without passing through the germinal center. Mateo et al. showed that only a minority of cases show *BCL6* somatic mutations [41]. Algara et al. also confirmed the molecular heterogeneity in this entity, with a group of SMZL that did not undergo somatic hypermutation and in which 7q31 deletions and shorter overall survival were more frequent [34].

#### 2.3.2. MicroRNA

Studies have highlighted the putative role of microRNA in the pathogenesis of these lymphomas and showed that the SMZL deregulates the expression of 51 miRNAs [26,42,43,44]. According to Arribas et al., the most overexpressed miRNAs seem to be “miR-155, miR-21, miR-34a, miR-193b and miR-100, while the most repressed miRNAs are miR-377, miR-27b, miR-145, miR-376a and miR-424” [42]. Focusing on miR-21-5p, its overexpression seems to be correlated with the aggressiveness of the disease [45]. Nevertheless, these findings seem not to be stackable with results observed in other lymphomas subtypes such as diffuse large B cell lymphomas (DLBCL), where miR-21 overexpression seems associated with a good prognosis [46,47].

Regarding the 7q32 deletion, Watkins et al. used qRT-PCR in SMZL with and without the 7q deletion to compare the levels of expression of numerous miRNA. They showed that miR593, miR-129, miR-182, miR-96, miR-183, miR-335, miR-29a, and miR-29b1 are consistently underexpressed in cases with 7q deletion as compared with those without the deletion [26]. In a single-cell analysis using magnetic cell separation, Arribas et al. also showed that miR-29b and miR-592 (located at 7q32) are repressed in SMZL B-cells compared with in healthy donors [42]. The arrival of these new data has made it possible to identify new potential target genes for the 7q deletion. For example, miR-29b is known to be involved in the oncogene T Cell Leukemia 1 Family AKT Coactivator A (*TCL1A*) upregulation [43]. However, Munari et al. evaluated *TCL1A* expression in a series of SMZL and showed *TCL1A* staining is negative in 24/31 cases of SMZL (77%). Another potential candidate is *CAV1* (caveolin-1), located at 7q31, which can act either as an oncogene (as described in bladder, thyroid, and esophageal cancers) or as a tumor suppressor gene (in ovarian, lung, and mammary tumors), depending on the tissue type or the microenvironmental influence [22]. Ruiz-Ballesteros et al. showed a decrease in *CAV1* expression using cDNA microarray expression profiling in a cohort of 44 SMZLs [23]. Two hypotheses could explain a loss of *CAV1* in SMZL, either a direct loss by deletion of the 7q31 region where this gene is located or indirectly via microRNA. Watkins et al. invalidated the first hypothesis, showing that the deleted 7q region is between 7q32 and 7q35, excluding the 7q31 region. This study leads to the second hypothesis, involving regulatory mechanisms beyond chromosomal loss. Among the miRNA with reduced expression in SMZL, nine can target *CAV1*, including miR-199a, miR-376, and miR-485, and may promote tumor progression by inducing the overactivation of transcriptional factors (*IGF1R* for miR-376a; *IKKβ* and NFκB pathway for miR-199a) [33]. The role of miR-485 is not yet evident, and this data have yet been confirmed.

So far, the gene targeted by the 7q deletion is unknown, and further studies are required to identify it and clarify the role of miRNA in SMZL.

#### 2.3.3. Mutational Landscape

**Figure 3:**
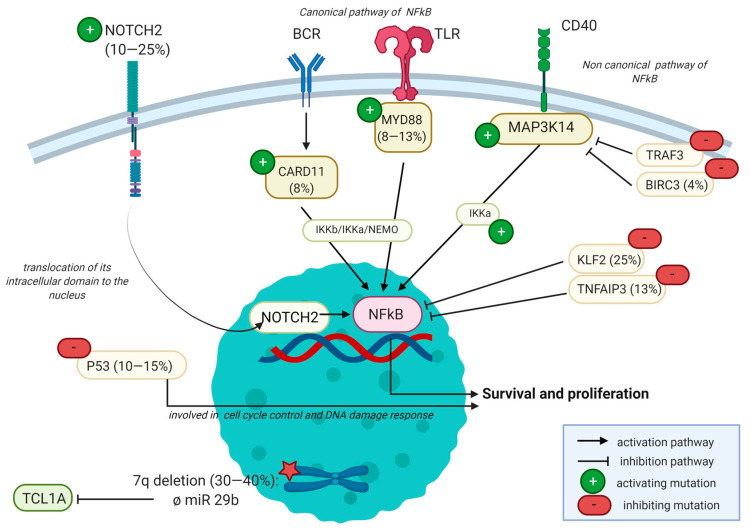
Summary of lymphomagenesis of SMZL created using BioRender.com thanks to these two reviews [48,50]. The mutation of *NOTCH2* (arising in 10–25% of SMZLs) will lead to the translocation of its intracellular domain to the nucleus, inducing a constitutive activation of this pathway and resulting in the transcription of multiple genes. It is associated with mutations that will have the effect of activating the NFκB pathway, such as *CARD11* (among 8% of cases) or *MYD88* via Toll-like receptors (TLRs). The canonical pathway for the NFκB activation also passes through *IKKb* (itself mutated in 4% SMZL). Other mutations impact the MAP3K14 pathway, involving *TRAF3* and *BIRC3* (mutated in 4% of SMZLs) complexes, leading to the activation of NFκB via its non-canonical pathway, passing through *IKKa*. Other mutations are inhibitory, such as the *KLF2* (25%) or *TNFAIP3* (13%) mutations. Apart from mutations, these two molecules play a role in inhibiting the NFκB pathway. Created with BioRender.com, accessed on 6 September 2021. Abbreviations: *BIRC3*, baculoviral IAP repeat-containing protein 3; *BCR*, B-cell receptor; *CARD11*, caspase recruitment domain-containing family member 11; *CD40*, cluster of differentiation 40; IGVH: immunoglobulin G heavy chain; *IKKa/b*, inhibitor of nuclear factor kappa B kinase subunit alpha/beta; *KLF2*, Krüppel-like factor 2; *MAPK*, mitogen-activated protein kinase; *MYD88*, myeloid differentiation primary response 88; *NEMO*, NF-kappa-B essential modulator; NFκB, nuclear factor kappa B; *NOTCH*, neurogenic locus notch homolog protein; *TCL1A*, T cell leukemia family AKT coactivator A; TLR, Toll-like receptor; TME, tumor microenvironment; *TNFAIP3*, tumor necrosis factor alpha-induced protein 3; *TP53*, tumor protein P53; *TRAF3*, tumor necrosis factor receptor-associated factor 3.

There are a growing number of studies describing the mutational landscape of SMZL [48]. Most recurrent mutations are *NOTCH2* (10–25% of cases), *KLF2* (20–30% of cases), *TP53* (10–15% cases), and mutations involving the NFκB pathway such as *CARD11, TNFAIP3, TRAF3*, or *BIRC3*. Active NFκB signaling is necessary for the generation and maintenance of normal marginal zone B-cells. Mutations involving the NFκB pathway concerned either the canonical pathway, passing by toll-like receptors such as *MYD88* or by a weak B-cell-receptor (BCR) signaling thanks to mutations of *CD79A/B, CARD11*, and/or *IKBKB*, or the non-canonical pathway with mutations of *TRAF3* or *BIRC3*, inducing CD40 signaling (Figure 3) [13,49,50]. The finality is the entry of the NFκB complex into the nucleus, where it can act as a transcriptional factor. Both canonical NFκB signaling and non-canonical NFκB signaling are deregulated. Another way to activate this pathway is by deletions or mutations of its negative regulator, encoded by the *TNFAIP3* (A20) gene, seen in almost 13% of SMZLs [51].

Mutations of *NOTCH2* occur in 10–25% of cases and mutations of *NOTCH1* in 5% of cases; these percentages are much lower in other subtypes of MZL [13,17,48,52,53,54,55,56]. The mutation of *NOTCH2* typically arises in the TAD and PEST domains and leads to constitutive activation of this pathway, resulting in the transcription of multiple genes. Mutations of negative regulators of *NOTCH* signaling (such as *SPEN, DTX1*, and *MAML2*) can also be seen, reinforcing the importance of this signaling pathway [13]. Moreover, *NOTCH2* mutations are associated with reduced treatment-free survival [57,58].

Mutations of *KLF2* occur in 12–44% of cases [13,59,60,61]. It is also a transcription factor of which the deficiency was previously shown to cause splenic marginal zone hyperplasia in mice. Its mutation leads to a translocation out of the nucleus in the cytoplasm, where it cannot inhibit the NFκB pathway. Clipson et al. demonstrated *KLF2* mutation distinguishes two subsets of SMZLs with distinct genetic changes. One subset with *KLF2* mutations for the 7q deletion rearranges IGHV1-2 with minimal somatic mutations with or without *KL2* mutations, but with pathogenic variants of *NOTCH2*, *TNFAIP3*, and *TRAF3* [59]. 

A mutation of *MYD88* that plays a significant role in the innate immune cells through Toll-like receptors (TLRs) can occur in 5% to 18% of SMZLs (mainly in those where plasmacytic differentiation is seen). They concern mainly the p.L265P variant (65% of cases) [57,62,63], but other variants of *MYD88* including p.V217F, p.M232T, and p.S219C have also been described [48]. *MYD88* mutation is also present in more than 90% of cases, raising the point of differential diagnosis. An isolated mutation of *MYD88* may not be discriminant; therefore, an isolated search for this mutation is not recommended in these cases. In the challenging diagnosis with LPL, the clinical presentation is of significant interest, with LPL being characterized by a medullar presentation with bone marrow involvement and an IgM monoclonal gammopathy. Splenic involvement is rare in LPL. The bone marrow’s involvement pattern is classically paratrabecular in LPL, whereas MZLs present mostly a nodular pattern [64]. Concerning molecular biology, the diagnosis is based on a set of arguments such as an association of *MYD88* L265P with *CXCR4*, *KNMT2D*, or *ARID1A* mutations, which are more in favor of an LPL [64,65,66]. *MYD88* is also affected by somatic mutations in 10% of NMZL and MALT lymphomas [13].

Mutations of *P53*, involved in cell cycle control, are described in 15% of cases and predominate at the DNA-binding domain [48]. These mutations seem to be associated with short overall survival [29,58,61]. In the study of Clipson et al., *MYD88* and *TP53* mutations are exclusively found in patients lacking *KLF2* mutations. Moreover, *MYD88* mutations and the 7q deletion seem mutually exclusive [51].

**Figure 4:**
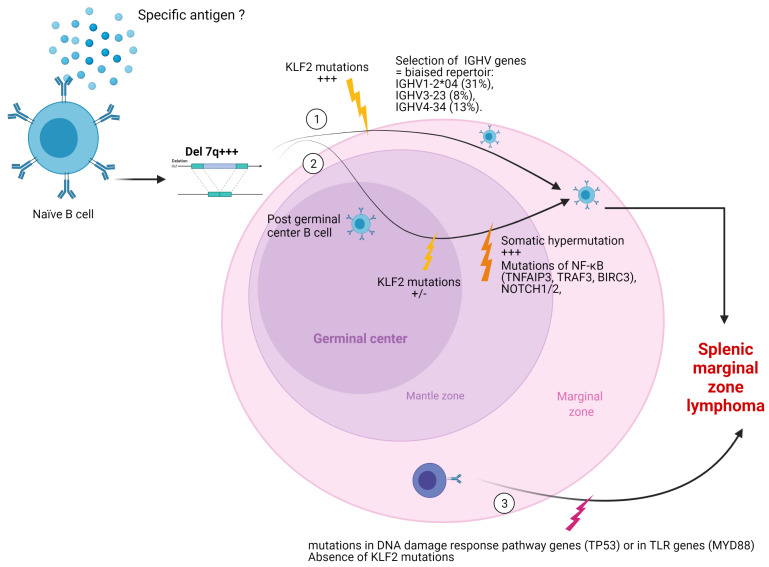
The top of the figure illustrates the two firsts pathways arising from a naïve B-cell and characterized by a deletion of the long arm of chromosome 7. Pathway n°1 is supposed not to pass through the germinal center. SMZL developed from this pathway presents a repertory bias with a preferential use of IGHV1-2*04 and is supposed not to undergo somatic hypermutation. Pathway n°2 is supposed to pass through the germinal center where somatic hypermutation happens, and mutations involving the non-canonical NFκB pathway and *NOTCH1/2* are added (sometimes associated with *KLF2* mutations). The third pathway is characterized by mutations of DNA repair genes (*TP53*) or TLRs genes (*MYD88*). More studies are required to precisely define these pathways and correlate them with histological patterns, immunohistochemistry, and clinical data (particularly prognosis). Created with BioRender.com. Abbreviations: *BIRC3*, baculoviral IAP repeat-containing protein 3; IGVH, immunoglobulin G heavy chain; *KLF2*, Krüppel-like factor 2; *MYD88*, myeloid differentiation primary response 88; *NOTCH*, neurogenic locus notch homolog protein; *TNFAIP3*, tumor necrosis factor alpha-induced protein 3; *TP53*, tumor protein P53; *TRAF3*, tumor necrosis factor receptor-associated factor 3.

As illustrated in Figure 4, the distribution patterns of genetic mutations suggest the possibility of two or three distinct pathways of SMZL lymphomagenesis.

*BIRC3* is also inactivated in SMZLs due to somatic mutations that disrupted its RING domain [49]. As described in other lymphomas such as mantle cell lymphomas [67], these genetic lesions activate the non-canonical NFκB pathway. In addition, its inhibition has also been associated with poor prognosis in chronic lymphocytic leukemia [68] and leads to fludarabine chemoresistance [69]. However, *BIRC3* belongs to the family of inhibitors of apoptosis proteins (IAPs) and has been designed to have a prosurvival and antiapoptotic role in solid and hematological tumors [70]. This antiapoptotic activity could be targeted using molecules such as second mitochondria-derived activator of caspases/direct IAP binding with low PI (SMAC) mimetics. The role of *BIRC3* warrants a more profound analysis in SMZL to clarify its role in these specific lymphomas.

As seen above, the differential diagnosis between the different subtypes of MZL can be challenging in cases of disseminated disease. They share common mutations, including those involving epigenetic regulation pathways (see below) or leading to an activation of the NFκB pathway, described in Table 1. In particular, somatic mutations or deletions of *TNFAIP3* arise in 18 to 29% of extranodal MZLs, 9% to 33% of NMZLs, and 8% of SMZLs [13,16,71]. However, each subtype also presents specific alterations that can be used for their differential diagnosis. Concerning SMZL, *KLF2* mutations are both relatively sensitive and specific markers of a splenic origin. They are indeed seen in 12% to 44% of SMZLs, only 9% to 20% of NMZLs, and 8% or 9% of MALT lymphomas [15,59,72]. To a lesser extent, mutations of the *NOTCH* pathway are also frequently described in SZMLs due to its role in homing B-cells to the splenic marginal zone [13]. Mutations of *NOTCH2* occur in 10% to 25% of SMZLs cases but are also seen in 20% to 25% of nodal MZLs [16,73] and 1.5% to 8% of extranodal MZLs [15,16,53,54,73]. *NOTCH1* mutations occur in 5% of SMZLs, but at a much lower frequency in NMZLs and not at all MALT lymphomas [74]. Conversely, NMZLs display an inactivation of *PTPRD* (14% in NMZLs vs. 3% in MALT lymphomas) and a much higher prevalence of mutations affecting *KMT2D* (34% in NMZL vs. 15% in MALT lymphomas) [13,16]. In MALT lymphomas, mutations of *TNFAIP3* and *CREBBP* (22% vs. 8%) are more frequent than in SMZLs [16]. Therefore, it is a set of arguments that can help differentiate the different forms of MZL.

#### 2.3.4. Epigenetic Regulation

However, regarding the consequences of those mutations, they are non-stereotypical and unsatisfactory as a therapeutic target. One other mechanism widely studied in B-cell lymphomas, but few in SMZLs, is post-transcriptional regulation via epigenetic modifications, such as the methylation of DNA promoter sequences. Indeed, mutations of genes implicated in epigenetic regulation such as *KMT2D* (*MLL2*, one of the most frequently mutated genes in NMZLs), *ARID1A, EP300, CREBBP*, and *TBL1XR1* are well known in other MZL, and some of them are described in SMZL too. These mutations are not discriminating in the definition of the origin of the lymphoma (nodal, splenic, and mucosal) [16]. Arribas et al. realized a clustering analysis of 98 SMZL and identified 2 clusters with different degrees of promoter methylation: the cluster with higher-promoter methylation, so-called “High-M,” has poorer overall survival compared with the lower-promoter methylation (“Low-M”) cluster [75]. Higher-promoter methylation is associated with mutations of the *NOTCH2* gene, 7q32 loss, and even histologic transformation.

### 2.4. Microenvironment

The importance of the microenvironment is well-known in other MZLs such as MALT lymphoma, which develops in response to chronic infection, inflammation, or autoimmune disease. Its development has been linked to the presence of pathogens such as Helicobacter pylori in gastric MALT lymphoma. In this disease, the infectious agent does not directly infect and transform lymphoid cells but rather chronically stimulates the immune system to maintain a protracted proliferative state, which indirectly increases the probability of lymphoid transformation. Helicobacter pylori indeed leads to the proliferation of marginal zone B-cells supported by T helper cells [76] and promotes an inflammatory environment in which neutrophils produce reactive oxygen species [77], all of which contribute to the acquisition of genetic aberrations by tumor cells.

Focusing on SMZL, hepatitis C virus (HCV) infection is a risk factor in this disease [78]. Moreover, in HCV-associated SMZLs, antiviral treatment (protease inhibitors, NS5A, or NS5B inhibitors) results in a marked reduction of lymphocytosis and splenomegaly. However, the underlying mechanisms are poorly understood [44].

These data illustrate the importance of focusing on the microenvironment to understand the lymphomagenesis of each lymphoma subtype. As far as SMZLs are concerned, few studies of this type exist at the moment.

Wickenden et al. performed multiplex semi-automated immunohistochemistry in a study including three SMZLs and found an essential role of T follicular helper (TFH) lymphocytes and regulatory T lymphocytes (Tregs) in the tumor microenvironment (TME) [79]. TFH cells are, indeed, significantly more often found near Ki67+ B-cells than Treg cells, suggesting for the first time that TFH cells might play a role in driving proliferation and hence contribute to the varying clinical course of MZL. This study demonstrates the necessity of a more profound understanding of immune effectors in SMZL TME. Other studies have concerned the innate immune system; one of them from Verney et al. identified distinct TLRs profiles in SMZLs [80]. The dense CD40 expression by bone marrow stromal cells (involving CD40 ligand expressed by mast cells) is correlated with a poorer prognosis [81]. The central hypothesis is that it happens through interactions of CD40 ligand with cells composing the TME (such as helper T cells, B-cells, macrophages, mast cells, or dendritic cells). The phosphorylation of STAT3 leads to the immune cell activation and secretion of IL-6 or TNF. These cytokines have the effect of enhancing tumor magnification and survival. Other cellular subtypes are less studied, such as neutrophils located around the marginal zone of the spleen, a B-cell area specialized in T cell-independent immunoglobulin responses to circulating antigen and act as B-cell helpers [82,83,84]. These neutrophils seem to influence the induction of immunoglobulin class switching, as well as somatic hypermutation or antibody production. They appear to activate B cells in the splenic marginal zone through a mechanism involving cytokines such as BAFF, APRIL, or IL-21. This particular type of neutrophils located in the splenic marginal zone could be present in the microenvironment of SMZL and play a role in their lymphomagenesis due to their ability to perform antigenic stimulation.

### 2.5. Limitations

As described above, there are many avenues of work in SMZL, but there are still few studies. The principal limit in the field of researches is the rarity of these lymphomas. Due to their low incidence, few human samples are available for research, all the more so with the increasingly less frequent use of splenectomy. Studies in murine or other animals models are also scarce [85], such as cell cultures that are very delicate in the field of lymphoma, all the more if indolent.

Moreover, many results are contradictory with other studies, as illustrated with miRNAs (e.g., miR-21, associated with a poor prognosis in SMZL, but with a good prognosis in DLCBL), or specific genetic mutations (e.g., *BIRC3* inactivation associated with poor prognosis in chronic lymphocytic leukemia [69], which is the contrary in other solid tumors or hematologic malignancies [70]).

### 2.6. Future Perspectives

#### 2.6.1. Future Studies

In the immune-checkpoint blockade therapies era, studies about the microenvironment appear essential to achieve in this disease. It is now accepted that the huge heterogeneity of immune infiltrates between and within solid tumor metastases, underlying the interest of considering the spatial organization of the whole tumor tissue to understand tumor/microenvironment crosstalk and predict response to treatment, in particular in the context of immunotherapy strategies. The microenvironment influences the survival and the proliferation of the tumor, either directly by promoting its development or because the tumor can acquire resistance mechanisms against its various components. One of the most studied mechanisms to do this is the escape from the immune system’s surveillance, particularly by slowing down T cells’ action. Numerous studies have been carried out in solid tumors to understand the link between tumor cells and their microenvironment [76,86,87,88,89,90]. Such studies have made it possible to develop monoclonal antibodies acting as inhibitors of “checkpoints” or immune control points and allowing “waking up” the immune system, such as ipilimumab, an antibody directed against the CTLA-4 protein (antigen 4 of cytotoxic T lymphocytes) [91,92]. The arrival of these treatments in the panel of available therapies has thus considerably improved the prognosis of melanoma.

These studies arrived much later in lymphomas, starting with classical Hodgkin lymphoma (cHL), in which results of immune-checkpoint blockade therapies are even better than in solid tumors, with moderate toxicity [86,93]. These clinical observations, coupled with important scientific discoveries, have uncovered salient features of the lymphoma microenvironment that correlate with immunotherapy response in patients. In small B-cell lymphomas such as follicular lymphomas, tumor cells also appear to depend heavily on the microenvironment for survival and proliferation [76]. For example, Carreras et al. have shown that numerous tumor-infiltrating lymphocytes (TIL) stained by PD1 before therapy by using a monoclonal antibody against PD-1 seem prognostically favorable [94]. Even if checkpoint blockade therapies are somewhat disappointing, for instance, in small B-cell lymphomas, these results suggest that studies focusing on the microenvironment could help develop such therapies in SMZL. Such meaningful approaches require state-of-the-art tools to analyze numerous parameters with high resolution in situ, a major technologically unmet need unsolved by classical single-cell RNAseq strategies. Among the available techniques, multiplex immunohistochemistry or immunofluorescence has emerged to be particularly promising, as illustrated by Wickenden and al. [79]. It provides high-throughput multiplex staining and standardized quantitative analysis for highly reproducible, efficient, and cost-effective tissue studies. Recent studies have demonstrated the interest in the technique of “GeoMx Digital Spatial Profiler (DSP)” to define the mechanisms of immunosuppression within the TME, in particular, to predict the response to anti-PD1 immunotherapies [95,96]. Such technologies open the field of possibilities to develop knowledge in these lymphomas and identify new therapeutic targets.

#### 2.6.2. Potentially Novel Therapeutic Targets

As described above, checkpoint blockade therapies are rather disappointing in small B-cell lymphomas [93,97,98]. However, few data focusing on marginal zone lymphomas, and in particular SMZLs, are available. Miller et al. described the case of a 77-year-old man with SMZLs (with a TP53 mutation) associated with metastatic melanoma. In this context, he benefited from treatment with pembrolizumab. Interestingly, the SMZL seemed to respond to this treatment, reducing spleen size, decreasing lymphocytosis and improving cytopenias [99]. Vincent-Fabert et al. also suggest that the PD-L1/PD-1 axis is effective in SMZLs [100]. They studied 54 SMZLs using immunohistochemistry and showed that, although tumoral cells are negative, PD-L1-positive cells are present in SMZL tumor nodules and associated with PD-1-positive cells and tend to be associated with shorter overall survival. However, here again, these data are controversial; another study, however, on a smaller cohort, did not show labeling with PDL-1 in the tumor cells or the microenvironment [101].

Some clinical trials test associations of pembrolizumab with idelalisib or ibrutinib (NCT02332980) in low-grade small B-cell lymphomas, particularly chronic lymphocytic leukemia also including SMZLs. Ibrutinib has proved its efficacy in CLL compared to placebo [102], and CLL is characterized by an immunosuppressive environment [103]. As previously shown, ibrutinib has also shown interesting results in relapsed or refractory SMZL, with an overall response rate of 62% [11].

A better understanding of these lymphomas’ pathophysiology and microenvironment could help develop new clinical trials using checkpoint blockade therapies and focusing on SMZLs.

Studies of the microenvironment in large cohorts could shed light on some of controversial biomarkers, some of which may also emerge as potential new therapeutic targets, such as the BIRC3 pathway using Smac mimetics (if its role was clarified) [70,104], or the protein NOTCH2 [105]. Concerning NOTCH2, some studies have tried to act on its ubiquitin-mediated proteasomal degradation, using proteasome inhibitors such as bortezomib. In CLL, this treatment has been found to reduce DNA/NOTCH2 complexes and cell viability. However, such treatments seem less effective on hematologic malignancies with a gain of function due to mutations of the PEST domain, such as SMZLs [73,105,106].

## 3. Conclusions

SMZL is becoming better characterized, notably on the histological and molecular levels. Studies have even hypothesized different lymphomagenesis pathways with different cytogenetic and molecular characteristics (including del 7q, IGVH repertoire bias, and KLF2 mutations) and potentially different prognoses. Despite this, it remains challenging to perform functional studies, and many questions about lymphomagenesis remain unanswered. It appears essential to improve our knowledge to ensure better patient management, because an unfavorable evolution requiring aggressive treatments is still often observed. Studies about the microenvironment seem particularly interesting in the new era of immunotherapy. Beyond the therapeutic consequences that such studies could have, the discovery of prognostic markers that could predict the evolution of patients at diagnosis is crucial.

## Figures and Tables

**Table 1:** Summarizing the principal SMZL biomarkers.

Biomarker	Role of the Protein	Consequence at Protein Level	Frequency	Literature
7q deletion	-	-	30%–40%	[26,29,30,31,32]
Trisomy 3	-	-	25%	[29,30]
Trisomy 12	-	-	10%	[29,30]
Trisomy 12	-	-	8%	
Preferential usage of specific IGHV genes:	-	-		[29,34,37,39]
IGHV1-2*04			31%	
IGHV3-23			8%	
IGHV4-34			13%	
*NOTCH2*	NFκB activation	Activation	10%–25%	[17,54,73]
*KLF2*	NFκB inhibition	Inactivation	12%–40%	[59,60,72]
*MYD88*	NFκB activation from the Toll-like receptors	Activation	5%–18% (p.L265P 65%)	[48,60,62,65,66]
*CARD11*	NFκB activation from the B cell receptors	Activation	5%–9%	[48,54,62]
*P53*	DNA damage and cycle cell control	Inactivation	15%	[29,52,61]
*BIRC3*	MAP3K14 inactivation	Inactivation by disruption of the RING domain	5%–11%	[49,54]
*TRAF3*	MAP3K14 inactivation	Inactivation	3%–10%	[49,54]
*MAP3K14*	NFκB activation	Activation	1%–8%	[49,54]
*SPEN*	Notch inhibition	repress Notch signaling	5%–10%	[48,54]
*TNFAIP3*	NFκB inhibition	Inactivation	7%–13%	[48,71]
*IKBKB*	NFκB activation	Activation	7%	[54]
*KMT2D*	Epigenetic regulation	Inactivation	9%–15%	[48,58]
*ARID1A*	DNA damage, cycle cell control, and epigenetic regulation	Inactivation	4%–6%	[58,61]
*EP300*	Epigenetic regulation	Inactivation	2%	[58]
*CREBBP*	Epigenetic regulation	Inactivation	5%	[58,61]
*TBL1XR1*	Epigenetic regulation	Inactivation	1%	[58]

Abbreviations: *ARID1A*, AT-rich interactive domain-containing protein 1A; *BIRC3*, baculoviral IAP repeat-containing protein 3; *CARD11*, caspase recruitment domain-containing family member 11; *CREBBP*, C-AMP response element-binding protein; *EP300*, E1A-binding protein P300; IGVH, immunoglobulin G heavy chain; *IKKa/b*, inhibitor of nuclear factor kappa B kinase subunit alpha/beta; *KLF2*, Krüppel-like factor 2; *KMT2D*, histone-lysine N-methyltransferase 2D; *MAPK*, mitogen-activated protein kinase; *MYD88*, myeloid differentiation primary response 88; *NFκB*, nuclear factor kappa B; *NOTCH*, neurogenic locus notch homolog protein; *TBL1XR1*, transducin (beta)-like 1 X-linked receptor 1; *TNFAIP3*, tumor necrosis factor alpha-induced protein 3; *TP53*, tumor protein P53; *TRAF3*, tumor necrosis factor receptor-associated factor 3.

## Data Availability

Data sharing not applicable and no new data generated.
